# Warfarin-related nephropathy: unveiling the hidden dangers of anticoagulation

**DOI:** 10.1007/s10238-024-01412-1

**Published:** 2024-07-03

**Authors:** Fengbo Xu, Guoqin Wang, Lijun Sun, Hong Cheng

**Affiliations:** Department of Nephrology, Beijing Anzhen Hospital, Capital Medical University, Beijing, China

**Keywords:** Warfarin-related nephropathy, Acute kidney injury, International normalized ratio, IgA nephropathy

## Abstract

Warfarin-related nephropathy (WRN) is defined as acute kidney injury subsequent to excessive anticoagulation with warfarin. Patients with mechanical prosthetic valves require long-term anticoagulant therapy. Nonetheless, warfarin remains the sole available option for anticoagulant therapy. Consequently, patients with mechanical prosthetic valves constitute a special group among the entire anticoagulant population. The present study recorded two cases of patients who had undergone mechanical prosthetic valve surgery and were receiving warfarin therapy. They presented to the hospital with gross hematuria and progressive creatinine levels. Notably, their international normalized ratio (INR) did not exceed three. Subsequent renal biopsies confirmed WRN with IgA nephropathy. The two patients continued to receive warfarin as anticoagulation therapy and were prescribed oral corticosteroids and cyclophosphamide, which resulted in improved renal function during the follow-up. Based on a review of all relevant literature and the present study, we proposed a new challenge: must elevated INR levels be one of the criteria for clinical diagnosis of WRN? Perhaps some inspiration can be drawn from the present article.

## Introduction

Warfarin-related nephropathy (WRN) is an increasingly recognized disease, mainly associated with excessive anticoagulation with warfarin. The preliminary description of WRN indicated that patients receiving warfarin therapy experienced significant glomerular hemorrhage and renal tubular obstruction by red blood cell (RBC) casts during renal biopsy [1]. WRN is defined as the manifestation of acute kidney injury (AKI) within a week after the international normalized ratio (INR) exceeds three [2].

Despite the development of direct oral anticoagulants (DOACs), research findings on the efficacy of DOACs in preventing valve thrombosis in patients with artificial valves have been inconsistent [3–5]. Therefore, warfarin remains the only available option for anticoagulants in this population. Patients who undergo mechanical artificial valve surgery are usually younger and have fewer comorbidities than those with atrial fibrillation, necessitating long-term anticoagulant therapy [6]. Accordingly, patients with mechanical prosthetic valves constitute a unique subgroup in the entire population of patients receiving anticoagulation therapy.

The present study reported two cases of patients with mechanical prosthetic valves who experienced gross hematuria and AKI. Ultimately, renal biopsy confirmed the diagnosis of WRN in these patients. However, an INR > 3 was not observed.

## Case presentation

### Case 1

A 56-year-old female patient, who had undergone mechanical aortic valve replacement surgery nine months ago and was prescribed warfarin as post-surgical treatment, was admitted to the hospital with gross hematuria, which started six days before hospitalization. Additionally, her creatinine level rapidly increased from 120.5 to 207.5 μmol/L within 3 days.

Upon admission, the patient’s vital signs were recorded as follows: blood pressure, 132/74 mmHg; heart rate, 83 beats/min; and temperature, 36.3 °C. Laboratory analysis revealed the following findings: creatinine level, 167.8 μmol/L;estimated glomerular filtration rate (eGFR), 30.5 mL/min.1.73 m^2^;blood urea nitrogen/creatinine ratio,17.9;albumin level, 39.6 g/L; hemoglobin level, 10.4 g/dL; white blood cell count,6.6 × 10^9^/L;platelet count,238 × 10^9^/L; an INR of 2.08. Urinary test results indicated the presence of urinary protein( +) and urinary occult blood(3 +). Microscopic examination of the urine sediment revealed 20–40 RBCs per high-power field, with a deformation rate of 70%. The quantification of urinary protein over 24 h was measured to be 0.69 g. Moreover, the *C*-reactive protein level was 8.49 mg/L (normal range, 0–5 mg/L). Other tests, including serum immunoglobulins (IgA, IgG, and IgM), antinuclear antibody, acute hepatitis panel, double-stranded deoxyribonucleic acid, rheumatoid factor, complement levels (C3 and C4), and antineutrophil cytoplasmic antibody profile, all yielded negative results. Serum protein electrophoresis and serum immunofixation also yielded negative results. Renal ultrasound examination indicated a right kidney size of 9.4 cm and a left kidney size of 9.9 cm, exhibiting normal cortical thickness.

**Fig. 1:**
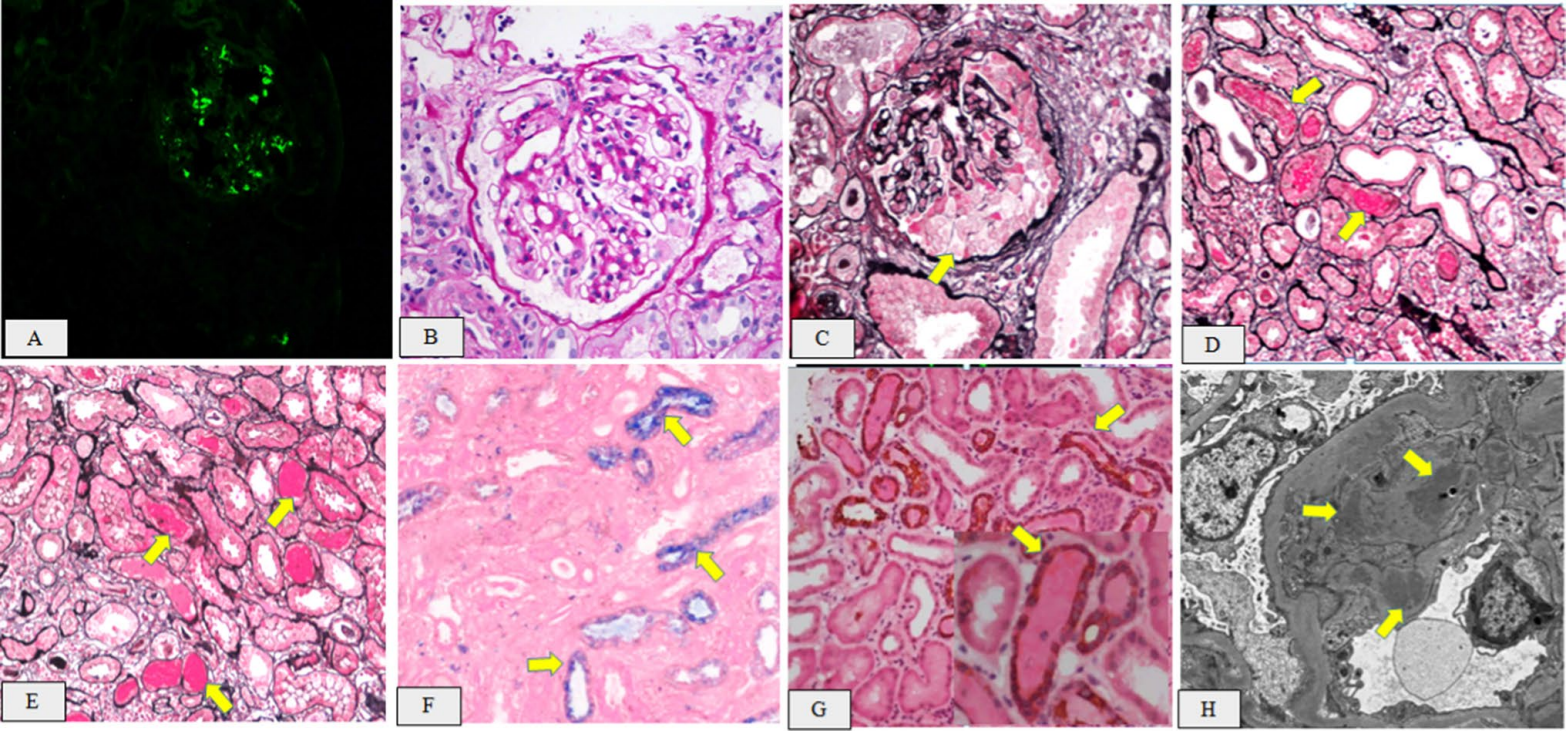
**A** Immunoglobulin A (IgA) deposition on the nephron(× 200). **B** Mild mesangial cell proliferation and increased mesangial matrix in the glomeruli(PAS × 400). **C** Signs of one large cell crescent(PASM + Mas × 200). **D**, **E** Signs of red blood cell casts in the tubules (PASM + Mas × 200). **F **Signs of hemosiderin deposits in tubular epithelial cells (Perl’s Prussian Blue stain × 400). **G** Immunohistochemical staining for cytokeratin AE1/AE3 highlighting the distal tubules with occlusive RBC casts (Hematoxylin and eosin [HE] × 200). **H** Numerous electron-dense substances are deposited within the mesangial region (EM × 6000)

A renal biopsy was performed on day 7 of admission. Immunofluorescence analysis demonstrated a 3 + intensity of IgA and 2 + intensity of C3, primarily displaying a coarse-grained pattern within the mesangial region. Light microscopy revealed that the renal tissue contained a total of 21 glomeruli. Among these, one glomeruli exhibited ischemic sclerosis, whereas two glomeruli displayed ischemic shrinkage. The remaining glomeruli showed mild mesangial cell proliferation and increased mesangial matrix. Furthermore, the renal capsule displayed one large cell crescent and one small fiber crescent.Moreover, renal interstitial fibrosis, accounting for approximately 35%, and renal tubular atrophy were observed. Tubular cross sections displayed a notable presence of RBCs casts. Perl’s Prussian Blue staining revealed hemosiderin deposits in tubular epithelial cells. Immunohistochemical staining for cytokeratin AE1/AE3 highlighted the distal tubules with occlusive RBC casts. Additionally, a mild thickening of the renal arteriolar wall was observed. Electron microscopy (EM) demonstrated a mild proliferation of glomerular mesangial cells and matrix, accompanied by the accumulation of clustered electron-dense substances within the mesangial region and the fusion of most epithelial foot processes (Fig. 1). Thus, the patient was diagnosed with WRN with focal proliferative IgA nephropathy.

**Fig. 2:**
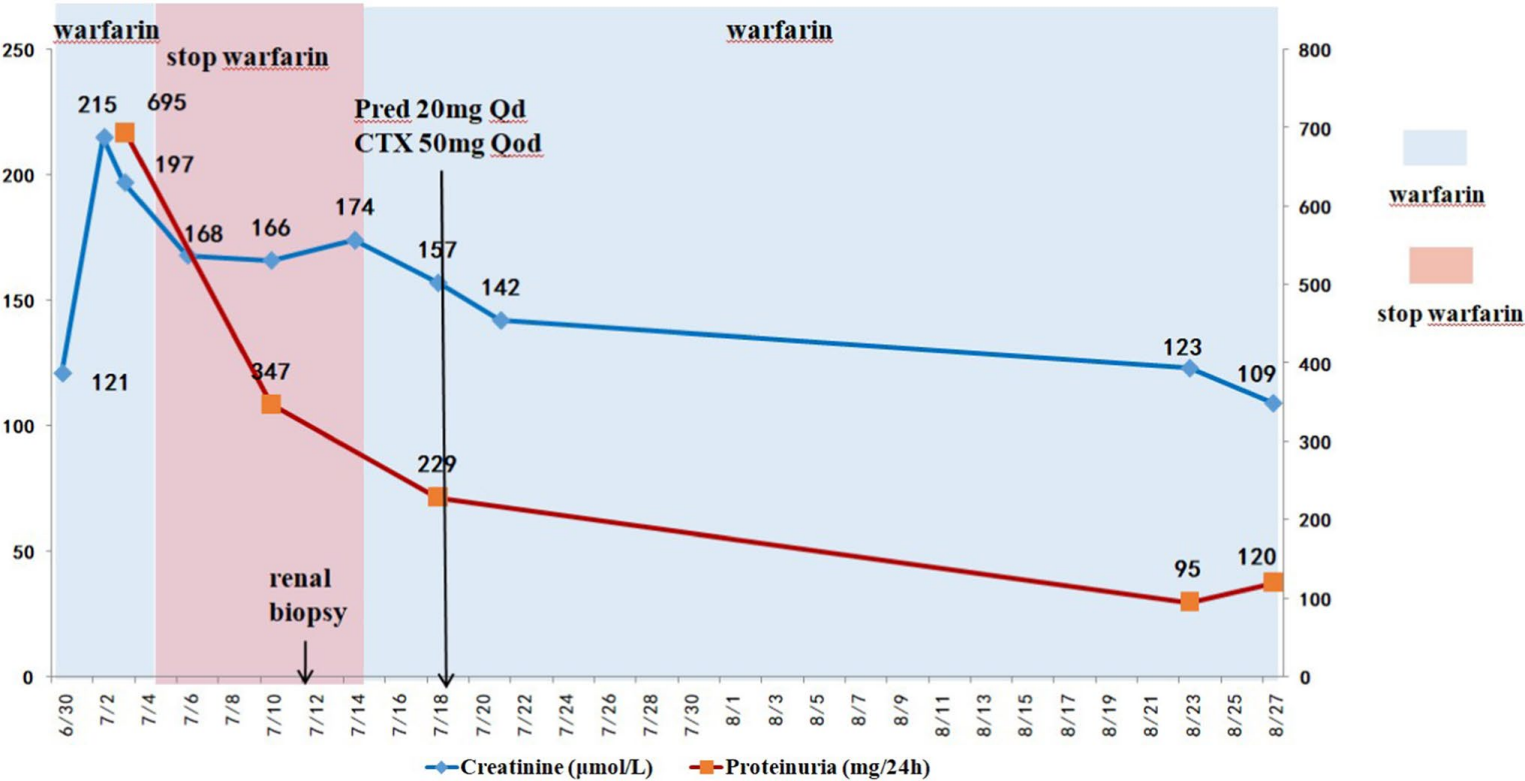
Clinical course in case 1

The patient continued taking warfarin anticoagulant and was prescribed a supplementary treatment regimen comprising oral sodium bicarbonate and prednisolone at a daily dose of 20 mg and cyclophosphamide at a dose of 50 mg every other day. Before discharge, the patient’s creatinine level was assessed to be 156.6 μmol/L. Upon follow-up after two months, the patient’s creatinine level decreased to 109.2 μmol/L, and the urine protein quantification was recorded as 119.7 mg/24 h (Fig. 2).

### Case 2

A 63-year-old male patient with a history of 20 years of hypertension was admitted to the hospital due to persistent gross hematuria for 40 days and an increase in creatinine levels for one month. Five years before, the patient had undergone partial nephrectomy for left renal clear cell carcinoma. Five and a half years ago, the patient underwent Bentall + Sun’s surgery involving aortic root replacement, total aortic arch replacement, and stent elephant nose surgery to manage type A aortic dissection. Following surgery, the patient was prescribed warfarin because of the replacement of a mechanical valve.

Upon admission, the vital signs were recorded as follows: blood pressure, 192/124 mmHg; heart rate, 76 beats/min;temperature, 36.3 °C. Laboratory analysis revealed a creatinine level of 169.9 μmol/L (which was within the normal range one year ago), an eGFR of 37.2 mL/min.1.73 m^2^, blood urea nitrogen/creatinine ratio of 14.1, albumin level of 39.5 g/L, hemoglobin level of 13.3 g/dL, white blood cell count of 6.27 × 10^9^/L, platelet count of 204 × 10^9^/L, and an INR of 2.69. The urinary test indicated the presence of urinary protein (2 +) and urinary occult blood (3 +). The microscopic analysis of the urine sediment revealed numerous RBCs in a high-power field, exhibiting an 80% deformation rate. The urinary protein quantification was 3.06 g/24 h. The serum IgA level was measured at 4.55 g/L, falling within the normal range of 1.0–4.2 g/L. Moreover, IgG and IgM levels were within normal limits. Results for antinuclear antibody, acute hepatitis panel, double-stranded deoxyribonucleic acid, rheumatoid factor, complement levels (C3 and C4), and antineutrophil cytoplasmic antibody profile were all normal. Serum protein electrophoresis and serum immunofixation results were negative. Renal ultrasound revealed a right kidney size of 11.8 cm and a left kidney size of 11.5 cm, demonstrating normal cortical thickness.

**Fig. 3:**
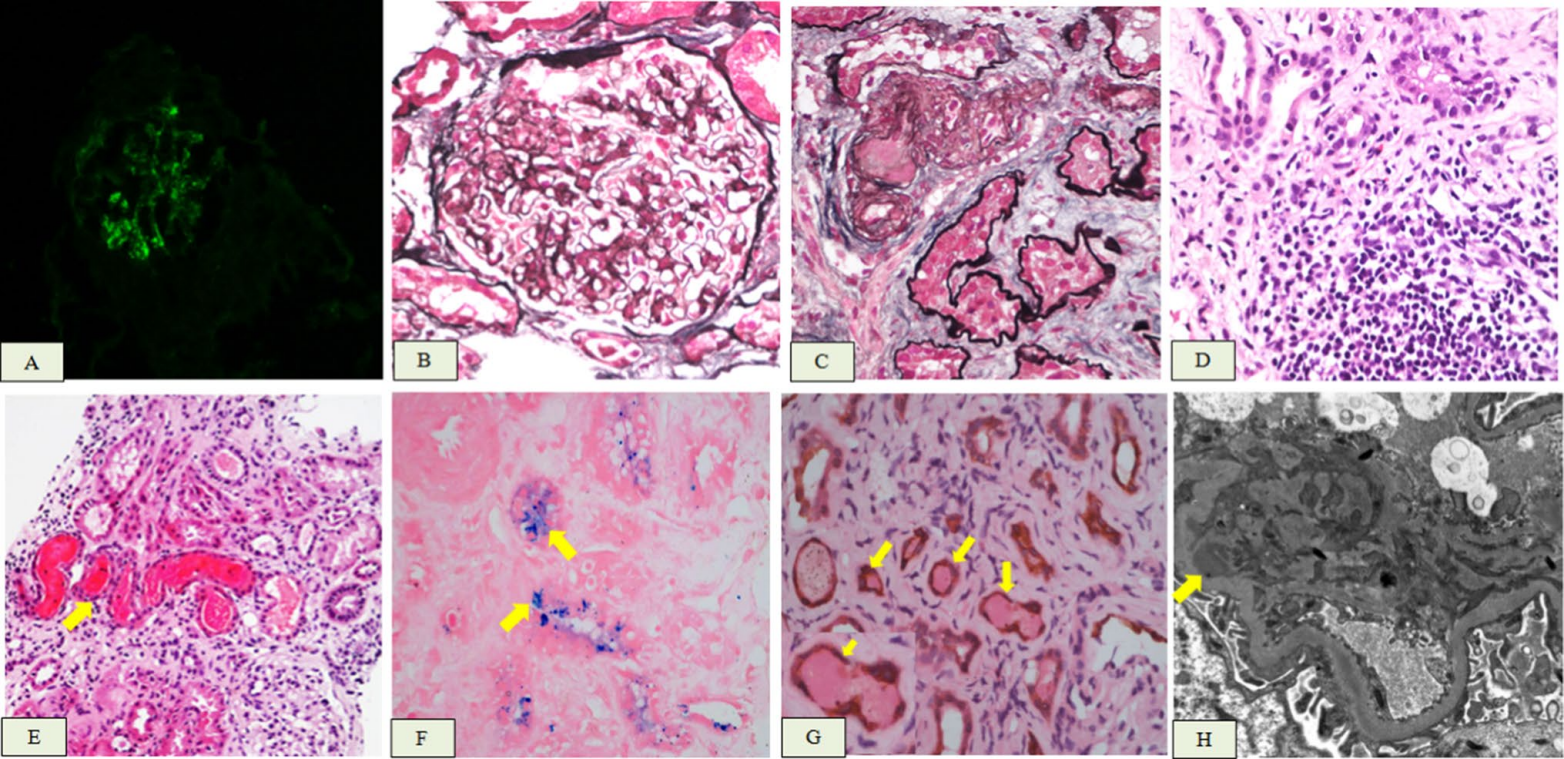
**A** Immunoglobulin A (IgA) deposition on the nephron(× 200). **B** Mild mesangial cell proliferation and increased mesangial matrix in the glomeruli(PASM + M × 400). **C** Thickening of the arteriolar wall and hyalinization of the intima (PASM + M × 400). **D** Multiple infiltration of mononuclear cells and occasional eosinophils and plasma cells in the renal interstitium(HE × 400). **E** Signs of RBC cast in the tubules(HE × 200). **F** Signs of hemosiderin deposits in tubular epithelial cells (Perl’s Prussian Blue stain × 400). **G** Immunohistochemical staining for cytokeratin AE1/AE3 highlighting the distal tubules with occlusive RBC casts(HE × 200). **H** Numerous electron-dense substances are deposited within the mesangial region (EM × 8000)

During hospitalization, the patient’s creatinine level reached a maximum of 231.4 μmol/L. A renal biopsy was performed on day 14 of admission. Subsequent immunofluorescence analysis demonstrated a 2 + intensity of IgA and C3 accompanied by segmental mesangial granular deposition. Light microscopy showed 21 glomeruli, of which approximately 33.2% (7 glomeruli) exhibited ischemic sclerosis, while approximately 14.3% (3 glomeruli) displayed ischemic shrinkage. The remaining glomeruli demonstrated mild mesangial cell proliferation and an increase in mesangial matrix, accompanied by segmental exacerbation and occasional endothelial cell proliferation. One glomerulus exhibited segmental sclerosis. Renal interstitial multifocal fibrosis, accounting for approximately 60%, was accompanied by a significant infiltration of mononuclear cells, occasional eosinophils, and plasma cells. Furthermore, granular and vacuolar degeneration in renal tubular epithelial cells and multifocal renal tubular atrophy were observed. Additionally, detachment of brush-like edges of renal tubular epithelial cells, flattened epithelial cells, and the presence of a large amount of RBC cases and a small amount of exfoliated cells in the lumen were noted. Moreover, there was evidence of epithelial cell regeneration along with the disappearance of normal structures in numerous renal tubules. Perl’s Prussian Blue staining revealed hemosiderin deposits in the tubular epithelial cells. Immunohistochemical staining for cytokeratin AE1/AE3 highlighted the distal tubules with occlusive RBC casts. The EM found mild mesangial cell proliferation and augmentation of the mesangial matrix within the glomerulus. The deposition of blocky electron-dense substances could be observed in the mesangial and paramesangial regions, along with the fusion of segments of the foot processes. An increase in lysosomes within renal tubular epithelial cells and partial detachment of microvilli were also noted. Finally, renal interstitial edema was detected (Fig. 3). The patient was diagnosed with WRN with focal proliferative IgA nephropathy.

**Fig. 4:**
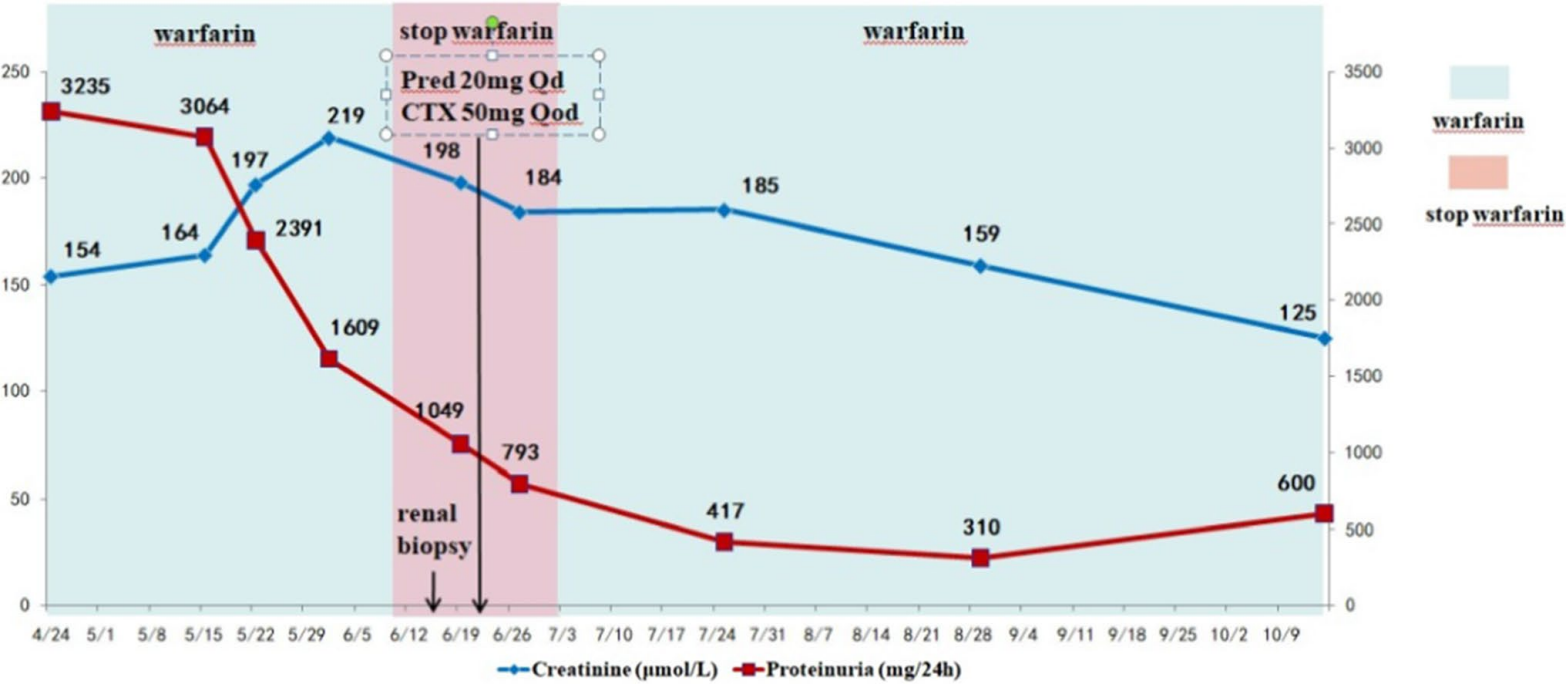
Clinical course in case 2

Anticoagulation therapy with warfarin was continued, along with a daily dose of 20 mg prednisolone and an oral dose of 50 mg cyclophosphamide every other day.Before discharge, the patient’s creatinine level was assessed at 213.5 μmol/L. During a subsequent follow-up after four months, the patient’s creatinine level decreased to 125 μmol/L, and the quantification of urine protein was recorded at 600 mg/24 h (Fig. 4).

## Literature review

In previous literature, ten cases of WRN confirmed by renal biopsy in patients with prosthetic valves were summarized [7–13]. The age of the patients ranged from 50–76 years, with a time interval of 11 months to 26 years from the initiation of oral warfarin administration to the onset of WRN. Six patients showed elevated baseline creatinine levels. The INR varied from 1.36–6.08. Two patients had an INR < 3 upon admission. Patients in all cases exhibited a progressive elevation in creatinine levels and the presence of hematuria. Among these cases, eight presented with gross hematuria, while the remaining two suffered from microscopic hematuria. Furthermore, six cases were complicated by IgA nephropathy. Steroids were administered in five cases. Dialysis treatment was initiated in four cases at the onset of WRN; three remained on dialysis, while one case experienced improved renal function and was no longer dependent on dialysis. Notably, three cases in which renal function did not recover had elevated baseline creatinine levels, ranging from 180–430 μmol/L. The remaining six cases showed varying degrees of improvement in renal function (Table 1).

**Table 1:** Clinical and demographic data of WRN patients with prosthetic valves

Patient no.	Country of author	Age (*y*)	Sex	Symptoms	Time to ARN onset	Surgery	INR (IU)	Baseline Scr (μmol/L)	Scr at Biopsy (μmol/L)	Renal biopsy pathology	Medications	Outcome
1 [7]	Slovenia	51	*M*	Gross hematuria	15 years	Aortic valve replacement	5.0	170–200	249	IgAN, ATI	Temporary stop warfarin; steroids	Incomplete recovery
2 [7]	Slovenia	76	*M*	Gross hematuria	10 years	Aortic valve replacement	4.4	430	487	global glomerular sclerosis, ATI	Dialysis; better control of INR	Dialysis
3 [7]	Slovenia	56	*F*	Microscopic hematuria	11 months	Aortic valve replacement	2.42	72	81	IgAN	Better control of INR	Stable kidney function
4 [7]	Slovenia	66	*M*	Gross hematuria	26 years	Aortic valve replacement	1.36	149	669	IgAN	Transiently converted to heparin	Kidney function slightly improved
5 [8]	Singapore	56	*F*	Microscopic hematuria	6 years	Valve replacement	4.95	72	317	IgAN	Prednisolone; acetylcysteine;	Incomplete recovery
6 [9]	India	50	*F*	Gross hematuria	2 years	Mitral valve replacement	4.70	80	415	acute tubulointerstitial nephritis	Steroids; temporary stop warfarin	Complete recovery
7 [10]	USA	57	*F*	Gross hematuria	26 years	Aortic valve replacement	3.71	274	518	diffuse mesangial proliferative glomerulonephritis ATI	Steroids; dialysis	Dialysis
8 [11]	Japan	55	*M*	Gross hematuria	13 years	Aortic valve replacement	3.75	67	796	IgAN	Supportive care	Incomplete recovery
9 [12]	Tailand	56	*M*	Gross hematuria	2 years	Aortic valve replacement	6.08	124	1017	ATI	Temporary stop warfarin; oral vitamin K; dialysis	Incomplete recovery
10 [13]	USA	61	*M*	Gross hematuria	available	Mitral valve replacement	3.52	180	601	IgAN	Steroids; dialysis	Dialysis
11*	China	56	*F*	Gross hematuria	9 months	Aortic valve replacement	2.08	121	215	IgAN, ATI	Steroids; cyclophosphamide	Incomplete recovery
12*	China	63	*F*	Gross hematuria	5.5 years	Aortic valve replacement	2.69	170	219	IgAN, ATI	Steroids; cyclophosphamide	Incomplete recovery

*SCr*, serum creatinine; *INR*, international normalized ratio; *RBC*, red blood cell; *IgAN*, IgA nephropathy; *LMWH*, low molecular weight heparin; *ATI*, acute tubular injury

^*^The present study

## Discussion

WRN is defined as AKI due to excessive warfarinization without evidence of clinically relevant hemorrhage [2]. The primary mechanism underlying AKI in WRN is the occurrence of glomerular hemorrhage and tubular obstruction caused by RBC casts [1]. Patients who have undergone mechanical artificial valve surgery require long-term anticoagulation treatment with warfarin. The pharmacokinetics of warfarin are influenced by various drugs and foods, and there is a potential risk of excessive warfarinization during long-term use. Patients with mechanical artificial valves have a high risk of “valve blockage” if warfarin is discontinued, which in turn limits the number of patients who can undergo renal biopsy when WRN is suspected in clinical practice. According to previous literature,warfarin can be substituted with DOACs once WRN occurs; however, guidelines do not recommend DOACs for patients with mechanical prosthetic valves [14]. Consequently, there are currently no alternative oral anticoagulants to substitute warfarin.

We reported two cases of gross hematuria and creatinine progression after long-term warfarin anticoagulant therapy in patients with mechanical valves. Renal biopsy revealed renal tubular RBC casts in both cases. The WRN was diagnosed based on typical clinical and pathological evidence. Regarding clinical characteristics, the two cases in the present study are similar to cases of WRN documented in the literature review [7–13]; the two patients also exhibited gross hematuria and creatinine progression. The WRN was initially characterized as AKI, which occurs following excessive anticoagulation, with INR > 3. However, the two patients in the present study did not show an INR > 3. In our literature review, two out of ten cases of WRN following valve replacement surgery exhibited an INR < 3.Previous studies have also confirmed WRN in patients with an INR of < 3 through renal biopsy [1, 15, 16]. Considering the lack of available INR levels before the AKI episode in these two patients, it is highly probable that the highest INR value was not detected. Consequently, when patients on long-term warfarin treatment exhibit both hematuria and AKI in clinical practice, the possibility of WRN must be considered regardless of whether the INR is < 3.

Both patients in this study had IgA nephropathy, which is consistent with the previous literature. A study in Spain involving 26 patients diagnosed with WRN reported that 73% of patients presented with IgA nephropathy [16]. A study of 41 WRN cases in the USA found that 43% of patients exhibited IgA deposition [17]. A retrospective study comprising 13 cases of WRN in Slovenia reported that 84.6% of the patients presented with IgA nephropathy [7]. Based on the evidence previously mentioned, it can be inferred that IgA nephropathy might be the predominant glomerular disease in patients with WRN. In clinical practice, IgA nephropathy is also characterized by hematuria and AKI, thus emphasizing the significance of renal biopsy as a definitive diagnostic tool for WRN.According to the findings in previous literature, except for IgA nephropathy, WRN frequently coexists with diabetic nephropathy, lupus nephritis, thin basement membrane nephropathy, and other glomerular diseases [1, 18, 19]. It is widely recognized that warfarin can decrease thrombin levels by inhibiting prothrombin synthesis. Thrombin, in turn, can bind and activate a family of proteinase-activated receptors (PARs) that are expressed in numerous cells, including glomerular endothelial cells [20]. It is hypothesized that activation of PARs provides trophic support to maintain endothelial integrity.Consequently, the decrease in thrombin levels induced by warfarin breaks down the endothelial barrier, thereby facilitating glomerular hemorrhage [21]. Patients with chronic kidney disease (CKD) of multiple etiologies frequently have a pathological foundation of glomerular endothelial injury, thereby significantly increasing the probability of WRN.Meanwhile, studies found that the transcription and concentration of cytochrome P450 enzymes were lower in CKD animal models, resulting in reduced drug metabolism and an increased risk of overdose [22]. This may also contribute to the heightened susceptibility of CKD patients to WRN.

Currently, there is a lack of established guidelines for treating WRN. The predominant strategies address coagulopathy and offer comprehensive supportive care.Most studies recommend modifying anticoagulant therapy from warfarin to NOACs [14]. For patients with mechanical prosthetic valves, NOACs are not recommended as per guidelines.In this study, both patients continued to receive anticoagulation therapy with warfarin and strengthened monitoring to ensure their INR remained within the range of three. Early administration of steroids has been reported to accelerate recovery from the classic inflammation observed in drug (anticoagulant)-induced nephritis [22–25]. Other studies have indicated that the etiopathogenesis of tubular injury associated with RBC casts can be ascribed to the detrimental local effects of catalytic iron released from decaying erythrocytes. The latter is thought to stimulate excessive production of hydroxyl radicals, damaging the lipoprotein components of tubular cell membranes and ultimately leading to apoptosis/necrosis of the tubular cells [26, 27]. Studies have demonstrated that corticosteroids can ameliorate associated tubulointerstitial injury and prevent progression to irreversible fibrosis. Theoretically, glucocorticoids may be a viable option for WRN therapy. Certain scholars have also highlighted that glucocorticoids are commonly used in anticoagulant-related nephropathy patients with potential IgA nephropathy and in patients with more prominent crescent lesions or tubulointerstitial inflammation. Such treatment aims to alleviate inflammatory responses and promote renal recovery [28]. Five out of the ten cases reviewed in the literature received glucocorticoids, four of them presented with immune nephritis, and the remaining displayed acute interstitial tubular lesions [7–10, 13]. According to the pathological findings in the present study, both patients exhibited typical RBC cases and acute renal tubular injury in WRN, along with IgA nephropathy. The first patient exhibited crescentic lesions, whereas proteinuria was measured at 3.1 g/24 h in the second patient. Therefore, we administered oral corticosteroids in combination with low-dose oral cyclophosphamide.

In the subsequent follow-up, the renal function of both patients demonstrated varying degrees of recovery. Three of the ten patients in our literature review required maintenance dialysis. Notably, the baseline creatinine levels of these three patients were higher than those of other patients [7–13]. In Brodsky’s study, including nine WRN patients, three patients who experienced complete renal function recovery exhibited a normal baseline creatinine, while four patients requiring maintenance dialysis displayed basal renal insufficiency, with two patients having a baseline eGFR below 30 mL/min. 1.73 m^2^. Owing to the limited availability of clinical studies with large sample sizes on WRN, it has only been reported in individual case reports on the relationship between the renal prognosis and the severity of tubulointerstitial lesions, and the impact of early administration of glucocorticoid therapy [7, 29]. This suggested that for treating WRN patients after valve replacement surgery, in addition to adjusting the dosage of warfarin and strengthening monitoring to control INR standards as much as possible, it is likely to consider the rational use of low-dose corticosteroid and immunosuppressants based on the results of renal biopsy. However, confirmation from studies with larger sample sizes remains required.

In conclusion, given the widespread use of warfarin in various medical conditions, clinicians must identify and diagnose WRN promptly. Clinicians should maintain a state of increased alertness regarding the potential occurrence of WRN in patients who exhibit hematuria and elevated creatinine levels while on warfarin therapy, even if their INR remains within the normal range. Considering that most WRN cases have an underlying glomerular disease (mostly IgA nephropathy), using corticosteroids and immunosuppressive drugs may be an attractive option, particularly in patients who are unable to replace warfarin with NOACs.

## Data Availability

The data are available from the corresponding author upon reasonable request.
